# Muscular Performance and Blood Pressure After Different Pre-Strength Training Strategies in Recreationally Strength-Trained Women: Cross-Over Trial

**DOI:** 10.3390/jcdd12010007

**Published:** 2024-12-26

**Authors:** Estêvão Rios Monteiro, Linda S. Pescatello, Luis Leitão, Marcelo José Colonna de Miranda, Paulo H. Marchetti, Michelle Ribeiro Novaes, Gleisson da Silva Araújo, Victor Gonçalves Corrêa Neto, Jefferson da Silva Novaes

**Affiliations:** 1 Postgraduate Program in Physical Education, Universidade Federal do Rio de Janeiro (EEFD/UFRJ), Rio de Janeiro 21941-599, Brazil; michellern@ufrj.br (M.R.N.); profgleisson@ufrj.br (G.d.S.A.); jeffsnovaes@ufrj.br (J.d.S.N.); 2 Postgraduate Program in Rehabilitation Science, Centro Universitário Augusto Motta (UNISUAM), Rio de Janeiro 21032-060, Brazil; 3 Undergraduate Program in Physical Education, IBMR University Centre, Rio de Janeiro 22631-002, Brazil; 4 Undergraduate Program in Physical Education, Centro Universitário Augusto Motta (UNISUAM), Rio de Janeiro 21041-020, Brazil; marcelocolonna@souunisuam.com.br; 5 Department of Kinesiology, University of Connecticut, Storrs, CT 06269, USA; linda.pescatello@uconn.edu; 6 Sciences and Technology Department, Superior School of Education of Polytechnic Institute of Setubal, 2910-761 Setúbal, Portugal; luis.leitao@ese.ips.pt; 7 Life Quality Research Centre (CIEQV), 2040-413 Leiria, Portugal; 8 Resistance Training Laboratory, California State University, Northridge, CA 91330, USA; paulo.marchetti@csun.edu; 9 Strength Training Laboratory (LABFOR), Physical Education College, Universidade Federal de Juiz de Fora, Minas Gerais 3986-3998, Brazil; 10 Undergraduate Program in Physical Education, Estácio de Sá University (UNESA), Rio de Janeiro 27515-010, Brazil; profvictorneto@gamaesouza.com; 11 Undergraduate Program in Physical Education, Centro Universitário Gama e Souza (UNIGAMA), Rio de Janeiro 22621-090, Brazil; 12 Undergraduate Program in Physical Education, Universidade Federal do Rio de Janeiro (UFRJ), Rio de Janeiro 23890-000, Brazil

**Keywords:** hemodynamic response, manual therapies, massage, resistance exercise, stretching

## Abstract

Background: This study aimed to investigate the acute effects of different pre-ST strategies on muscular performance and blood pressure (BP) responses in recreationally strength-trained women. Methods: Twelve overweight women with normal BP were recruited and performed six experimental protocols in a randomized order: (1) control protocol (CC), where BP was assessed without exercises performed; (2) ST; (3) foam rolling warm-up followed by ST (FR + ST); (4) specific warm-up followed by ST (SW + ST); (5) aerobic exercise followed by ST (AE + ST); and (6) stretching exercises followed by ST (SE + ST). ST consisted of three sets at 80% of 10 repetition maximum with a self-suggested rest interval between sets for bench press, back squat, bench press 45°, front squat, lat pull-down, leg press, shoulder press, and leg extension. Results: All experimental protocol had a lower total training volume, fatigue index, and repetitions performance in relation to ST (*p* < 0.05). No significant reduction was observed in systolic and diastolic BP for any protocol or exercise, although the effect size magnitudes ranged from trivial to large. Decreases in maximum repetitions, resistance to fatigue, and total training volume were performed before ST as warm-up strategies. However, these strategies indicated a clinical reduction in BP with a large and meaningful magnitude (effect size) in recreationally strength-trained women with normal to elevated BP. Conclusions: The results of this investigation may help to influence decision-making by practitioners who desire to elicit a post-exercise hypotension response in both subjects with normal BP and hypertension.

## 1. Introduction

Regular physical exercise represents a significant non-pharmacological approach to promoting health and enhancing physical performance [1]. Notably, when analyzing the American College of Sports Medicine Worldwide Survey of Fitness Trends for 2024 [2], it becomes evident that 8 out of the 10 major trends are closely linked to physical exercise. This trend aligns with the recommendations previously set forth by the World Health Organization, which was recently updated to encourage individuals to engage in 300 min of physical activity per week.

In a study with a similar design to our current investigation, Da Silva Telles et al. [3] explored various warm-up strategies [including strength training (ST) with ischemic preprotocoling, specific warm-up (SW), aerobic exercise (AE), and dynamic stretching] prior to two strength training exercises (bench press and leg press 45°). The authors observed improvements in muscular performance, specifically in terms of maximum repetition performance and total training volume, when utilizing the pre-ST strategy with ischemic preprotocoling. The findings from Da Silva Telles et al. [3] and the study’s overall design have highlighted important research gaps, including the investigation of foam rolling (FR) or stretching as alternative pre-ST warm-up strategies. Notably, to the best of our knowledge, no prior studies have explored the effects of FR as a pre-ST strategy on blood pressure (BP) and muscular responses (total training volume, maximum repetition performance, and fatigue index). Further research in this area could provide valuable insights into optimizing warm-up techniques in strength training settings.

With approximately 31.1% of adults worldwide in the last decade experiencing hypertension, the influence of ST exercise on BP is an important health consideration [4]. Hypertension is considered a major risk factor for cardiovascular diseases [5], generating worldwide concern [6]. Post-exercise hypotension (PEH) is characterized as an immediate exercise-induced reduction in BP of 5–8 mmHg on average [7], a magnitude that has important clinical relevance to reducing cardiovascular risk throughout the day [7]. Several non-pharmacological strategies have been tested to promote PEH [7]. ST has recently been recommended as a non-pharmacological strategy in and of itself, with several studies indicating its effectiveness in promoting PEH in adults with and without hypertension [6], whether by manipulating the exercise order, rest interval, and training methods.

Although not as extensively studied, similar PEH effects are observed when using FR [8,9] and stretching [10] in subjects with normal BP. However, conflicting reports indicate that the concurrent sequencing of stretching and ST can impair, improve, or have no effect on performance (e.g., total training volume, force output, hypertrophy). Furthermore, there are limited studies regarding BP responses to different intervention combinations (e.g., FR, SE, aerobic exercise (AE), and ST). It is known that in relation to FR and SE, the mechanical stimulus during exercise triggers the pressor reflex that increases sympathetic discharge through efferent stimuli coming from control centers located in the central nervous system, such as the nucleus of the solitary tract [11,12]. Thus, considering that the neural mechanisms that partly explain PEH are related to a flattening of the post-exercise sympathetic stimulus as a compensatory means of sympathetic activity during exercise, it is plausible to hypothesize that such strategies may interfere in the PEH [13]. Vasodilator responses and reductions in cardiac output have been appointed as mechanisms for PEH caused by AE [14]. Thus, considering that the strategies employed in the present study have an impact on BP responses, investigating possible congruences that attenuate the post-exercise BP response is a knowledge gap that should be investigated further.

Thus, the purpose of the present study was to examine the acute effects of different pre-ST strategies on total training volume, maximum repetition performance, fatigue index, and BP responses in recreationally strength-trained women. The present study had two initial hypotheses. First, isolated ST may show better muscular performance compared to other pre-ST strategies. Second, the combination of FR or stretching with ST has been shown to potentially reduce BP values.

## 2. Materials and Methods

### 2.1. Ethical Considerations

The procedures of the present study were carried out in accordance with Resolution No. 466/12 of the Brazilian National Health Council. The study was submitted and approved by the Augusto Motta University Centre ethics committee [4.611.983] and was conducted in accordance with the Declaration of Helsinki.

### 2.2. Participants

Twelve recreationally strength-trained (resistance training experience: 29.8 ± 7.6 months) women with normal (baseline systolic BP: 118.33 ± 4.89 mmHg; baseline diastolic BP: 78.50 ± 4.10 mmHg) BP (age: 27.2 ± 3.3 years; height: 164.8 ± 5.5 cm; body weight: 69.8 ± 6.0 kg; body mass index: 25.7 ± 2.3) were recruited for the present study based on a priori sample size calculation [15]. An a priori sample calculation, based on BP values from the pilot collection (effect size = 0.65; 1-β = 0.95; α = 0.05; non-sphericity correction = 1.0), was performed using the G*Power software (v. 3.1.9.6) [16], indicating that twelve subjects would be adequate to achieve the aforementioned statistical power. Inclusion criteria were as follows: (1) Subjects had to participate in a structured ST program for at least one year prior to the study. The ST program had to average at least 50 min per session and consist of at least three sessions per week, using loads between 8 and 12 RM and rest intervals between 1 and 3 min between sets and exercises. (2) Subjects had to be free from any functional limitations or medical protocols that could have compromised their health or confounded the study results.

During the seven weeks of data collection, the subjects were instructed not to engage in any non-study ST program or other strenuous physical activity. Subjects were recommended to maintain their eating and sleeping habits, avoiding caffeine and ergogenic, over the seven weeks of data collection. Prior to the study, all subjects were provided with verbal and written explanations of all study procedures.

### 2.3. Procedure

**Figure 1:**
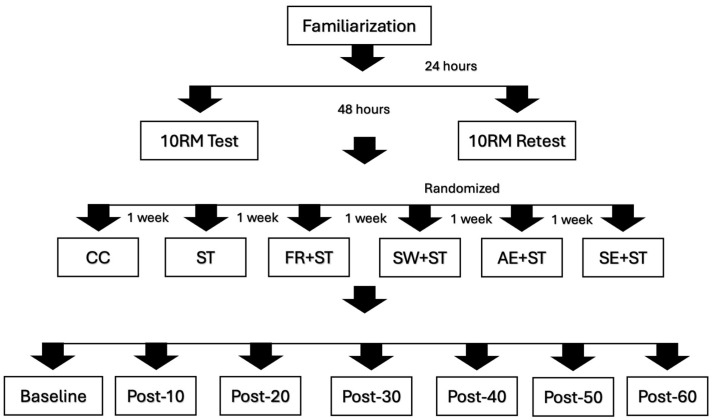
Study design. CC = control condition; ST = strength training isolated; FR + ST = foam rolling warm-up followed by strength training; SW + ST = specific warm-up followed by strength training; AE + ST = aerobic exercise warm-up followed by strength training; SE + ST = stretching exercise warm-up followed by strength training.

A randomized and counter-balanced within-subject experimental design was used to examine the acute effects of different pre-ST strategies on total training volume, maximum repetition performance, fatigue index, and BP responses in recreationally strength-trained women. Subjects visited the laboratory for nine sessions during a seven-week period with at least forty-eight hours and one week between visits for ten repetition maximum tests (10 RM) and each experimental protocol, respectively (see Figure 1).

The first session was used to familiarize subjects with all procedures. Sessions 2 and 3 were used for 10 RM testing and re-testing, respectively, for bench press, back squat, bench press 45°, front squat, lat pull-down, leg press, shoulder press, and leg extension exercises to determine the exercise load to be used during all experimental protocols. Following 10 RM testing, six experimental protocols followed in a randomized order: (1) control protocol (CC), where BP was assessed without exercises performed; (2) ST isolated (ST); (3) FR warm-up followed by ST (FR + ST); (4) SW warm-up followed by ST (SW + ST); (5) AE warm-up followed by ST (AE + ST); and (6) SE followed by ST (SE + ST). As a standardization measure, a passive interval of 5 min was adopted between the experimental warm-up conditions (FR, SW, AE, and SE) and the ST session. The total training volume (repetition × load) was recorded following each exercise and protocol.

During each experimental session, BP was assessed after a 15 min passive rest period on arrival at the laboratory and at 10, 20, 30, 50, and 60 min following the interventions (Baseline, Post-10, Post-20, Post-30, Post-40, Post-50, and Post-60). All procedures were performed at the same time (in the morning) to avoid any confounding circadian rhythm effect. Subjects were instructed to maintain their regular respiratory pattern to avoid the Valsalva maneuver.

### 2.4. Load Test

For the ten repetition maximum test (10 RM), the exercises performed in the 10 RM test were bench press, back squat, bench press 45°, front squat, lat pull-down, leg press, shoulder press, and leg extension. Briefly, subjects initially performed a standardized warm-up consisting of 15 repetitions with a self-suggested load (representing approximately 50% of the normal training load). Following the warm-up, 10 RM testing was performed for all exercises on the same day and in the same order with 10 min rest intervals between exercises. Execution of the exercises was standardized insofar as no pauses were allowed between concentric and eccentric portions of the lift. Repetitions were conducted at a constant velocity of 4 s per repetition (2 s concentric and 2 s eccentric) controlled by a metronome (Metronome Plus 2.0; M&M System, Lich, Germany). A maximum of three trials were allowed per testing session, separated by 3 min of passive rest. Testing was then repeated on another day at least 48 h later (re-test). The greater load between the two testing days was deemed the 10 RM load2.5. Instruments. Load reproducibility between the two testing days was tested by the intraclass correlation coefficient. To minimize potential error variance, the following procedures were adopted for all subjects, for they (a) received standardized instructions about the exercise technique and data collection, (b) received feedback regarding technique and were corrected when appropriate, and (c) were always verbally encouraged. The same exercise apparatus was used for 10 RM testing and during all strength training experimental sessions (ST, FR + ST, SW + ST, AE + ST, and SE + ST).

### 2.5. Instruments

During the strength training (ST) protocol, subjects performed all exercises until concentric failure throughout three sets of bench press, back squat, bench press 45°, front squat, lat pull-down, leg press, shoulder press, and leg extension exercises at 80% of their previously established 10 RM, with a self-suggested rest interval between sets. The number of repetitions in each set was recorded for each protocol.

Foam rolling (FR) protocol utilized a foam roller with a hard inner core enclosed in a layer of ethylene-vinyl acetate foam (Trigger Point Technologies, 5321 Industrial Oaks Blvd., Austin, TX 78735, USA), which has been shown to produce more pressure on the soft tissue than a conventional foam roller without a hard core. The FR was applied, unilaterally in a randomized order, in single sets of 90 s to the lateral torso of the trunk, anterior (i.e., quadriceps) and posterior (i.e., hamstrings) thigh, and calf regions. The lateral torso of the trunk was rolled in a decubitus lateral position. Subjects were instructed to roll their lateral aspect of the trunk up and down on the foam roller between the proximal third of the arm and the inferior part of the ribcage. Anterior and posterior thigh positions were performed in a plank and seated position, respectively, with the upper thigh of the dominant leg on the foam roller [17]. While keeping the knee of the dominant leg extended, subjects were instructed to use their arms and a contralateral leg to propel themselves backward and forward on the foam roller between the acetabulum and quadriceps tendon and between the ischial tuberosity and popliteal fossa for the anterior and posterior thigh, respectively, with fluid dynamic motions. The posterior leg was performed in a seated position with the legs extended and the feet relaxed. One leg was crossed over the other to allow more pressure to be directed over the plantar flexors being treated. Subjects were instructed to use their arms to propel their body back and forward, from the popliteal fossa to the Achilles tendon, in fluid motions. Subjects were encouraged to support as much of their entire body mass as possible with the foam roller, thus maximizing pressure on the limb. For a better representation of typical or realistic training environments, subjects were free to choose the pace with which they performed the roller. FR was applied at different angles to target all areas with controlled pressure through a pain level scale, in which a score of one represented no pain at all and a score of 10 represented maximal tolerable pain. Subjects were instructed to maintain pressure resulting in a self-rated score of 6 out of 10 on the pain level scale.

For each stretching exercise (SE), the joint movement was taken into a position of slight discomfort [1], and then, the joint position was maintained. Subjects were instructed to maintain their usual respiratory pattern throughout all SEs. Static stretching was applied unilaterally in a random order, with a single set for 90 s to the lateral torso of the trunk, anterior (i.e., quadriceps) and posterior (i.e., hamstrings) thigh, and calf regions. The lateral torso of the trunk static stretching was performed in a seated position with the stretched lateral torso flexed and non-stretched relaxed in extension. The therapist performed the movement of lateral torso flexion with the knee flexed to maximize stretching of the lateral torso of the trunk. Anterior thigh static stretching was performed in decubitus lateralis with the stretched leg flexed and non-stretched relaxed in extension. The therapist performed the movement of knee flexion with hip extension to maximize stretching of the anterior thigh muscles. Posterior thigh static stretching was performed in a supine position with the stretched leg extended and the non-stretched leg relaxed and flexed at 45° of hip and knee flexion. The therapist performed the movement of hip extension with an extended knee. The participants engaged in active posterior leg static stretching while standing. They positioned one leg on the edge of a bench, extended the knee, and dorsiflexed the ankle, directing the heel toward the ground. During the stretch, subjects leaned on the wall for balance if needed. Static stretching was performed passively with the dominant leg in the stretching position. The rating perceived was controlled by a self-rated pain pressure level scale of 6 out of 10.

The aerobic exercise (AE) protocol consisted of walking on the treadmill at a light to moderate self-regulated intensity between 30 and 60% of the heart rate reserve (HRR = (HR maximum − % intensity) + HR) for 12 min [3].

The specific warm-up (SW) was composed of two sets of 15 repetitions with 40% of 10 RM for bench press, back squat, bench press 45°, front squat, lat pull-down, leg press, shoulder press, and leg extension exercises. There was a 90 s rest interval between sets [18].

### 2.6. Measures

Systolic and diastolic blood pressure (BP) were measured using an automatic oscillometric device (Omron Hem 7113, São Paulo, Brazil) [19]. During each experimental session, blood pressure was assessed after a 15 min passive rest period on arrival at the laboratory and at 10, 20, 30, 50, and 60 min following the interventions (Baseline, Post-10, Post-20, Post-30, Post-40, Post-50, and Post-60). Measurements were performed on the left arm.

For muscular response, the total training volume (repetition × load) was recorded following each exercise and protocol. The fatigue index was analyzed from the first (1st) and last (3rd) sets and calculated using the equation [19] Fatigue index = (third set ÷ first set) × 100, where a higher percentage value (%) indicates a superior fatigue resistance. The value was noted at the end of each exercise set.

### 2.7. Statistical Analyses

Based on the sample size, the Shapiro–Wilk normality test was performed, followed by the symmetry and kurtosis analysis. Finally, the graphical distribution analysis with the histograms and QQ plot observation was tested. Variables that had their normality violated were described using the median and the interquartile range as measures of central tendency and descriptive statistics dispersion, which indicates the variability of results, respectively. For the variables that did not have their normality rejected, descriptive variables were calculated as the mean as a measure of central tendency as well as the standard deviation as a measure of the tendency of variability.

The total training volume had normality partially rejected, so the comparison between the protocols was made using the Friedman test (all exercises except bench press 45o) and a two-way ANOVA (8 exercises*5 protocol) with repeated measures (normal distribution for bench press 45o) for non-parametric and parametric tests to determine between and within effects of total training volume for each exercise and protocol. Pairwise comparisons adjusted by Bonferroni corrections were employed to score within protocol differences and the Wilcoxon test between protocol. Maximum repetition performance and fatigue index did not achieve normality, so the comparison between protocol and sets was made using the Friedman non-parametric test followed by a pairwise comparison adjusted by Bonferroni corrections. Blood pressure responses were normally distributed, so the comparison between protocol was made using the ANOVA (8 exercises*6 protocols) with repeated measures followed by Bonferroni post hoc test if any statistical difference was evident.

The interquartile range was calculated based on dividing a data set into quartiles and subtracting the third quartile (upper limits) from the first quartile (lower limits). Additionally, Cohen’s d effect sizes were calculated using the formula d = Md/Sd, where Md is the mean difference and Sd is the standard deviation of differences. This calculation differs slightly from traditional Cohen’s d calculations, in that this formula better represents within-subject differences, whereas the traditional Cohen’s d formula is better for between-subject comparisons [20,21,22,23]. Cohen’s d effect-sizes were defined as small, medium, and large for 0.2, 0.5, and 0.8, respectively.

An alpha level of 0.05 was used. All analyses were performed using SPSS version 21 (SPSS Inc., Armonk, NY, USA).

## 3. Results

Regarding the descriptive characteristics and reproducibility of the 10 RM test, subjects’ load characteristics were provided in Table 1. All exercises presented intraclass correlation coefficient values above 0.5, indicating moderate reproducibility (Table 1).

### 3.1. Total Training Volume

With all exercises, ST consistently demonstrated the highest total training volume. A significantly (*p* < 0.001) greater bench press total training volume was achieved with the ST over FR + ST, SW + ST, and SE + ST. Similarly, the back squat total training volume was significantly (*p* < 0.001) higher with ST versus FR + ST and SW + ST protocols. With the front squat, ST demonstrated significantly more (*p* < 0.001) total training volume than FR + ST, AE + ST, SW + ST, and SE + ST protocols. For the lat pull-down, ST significantly (*p* < 0.001) exceeded FR + ST, SW + ST, SE + ST, and AE + ST. For the leg press, ST was significantly (*p* < 0.001) greater than FR + ST, SW + ST, SE + ST, and AE + ST. For the shoulder press, ST was significantly (*p* = 0.002) higher than FR + ST, SW + ST, and AE + ST. For the leg extension, ST was significantly (*p* < 0.001) higher than FR + ST and SW + ST. Finally, for the bench press 45°, ST demonstrated significantly (*p* < 0.001) higher total training volume than FR + ST, SW + ST, and AE + ST. The only significant difference that did not involve the ST protocol showed that the bench press 45° with SE + ST exhibited a higher total training volume than FR + ST (all interaction details illustrated in Table 2).

### 3.2. Maximum Repetition Performance

There was a significant reduction in the maximum repetitions between sets 3 and 1 with the FR + ST protocol (bench press: *p* = 0.003; back squat: *p* < 0.001; bench press 45°: *p* < 0.001; front squat: *p* < 0.001; lat pull-down: *p* < 0.001; leg press: *p* = 0.018; shoulder press: *p* = 0.002; leg extension: *p* = 0.007). Similarly, significant reductions in the maximum repetitions were observed between sets 2 and 1 (bench press: *p* = 0.024 and shoulder press: *p* = 0.024) as well as between sets 3 and 1 (bench press: *p* < 0.001; back squat: *p* = 0.032; bench press 45°: *p* = 0.032; front squat: *p* = 0.007; lat pull-down: *p* = 0.009; leg press: *p* = 0.001; shoulder press: *p* = 0.002; leg extension: *p* = 0.002) with the SW + FR protocol. Furthermore, AE + ST presented significant reductions in maximum repetitions between sets 3 and 1 for bench press (*p* = 0.043), front squat (*p* = 0.002), leg press (*p* = 0.043), shoulder press (*p* = 0.009), and leg press (*p* = 0.013) exercises. Finally, significant reductions in the maximum repetitions were observed in the SE + ST protocol when comparing the third and second sets in the front squat exercise (*p* = 0.032) and between the third and first sets in the bench press (*p* = 0.024), bench press 45° (*p* = 0.032), front squat (*p* = 0.009), lat pull-down (*p* = 0.009), leg press (*p* = 0.005), shoulder press (*p* = 0.024), and leg press (*p* = 0.007) exercises. No other significant within-protocol differences were observed (all details presented in Table 3).

In the comparison between the experimental protocols, the bench press exercise presented a higher number of repetitions with set 2 in the ST protocol when compared to SW + ST (*p* = 0.006), SE + ST (*p* = 0.024), and FR + ST (*p* = 0.030) as well as in set 3 when comparing SW + ST (*p* < 0.001) and FR + ST (*p* < 0.001) protocols. Likewise, a greater number of repetitions was performed in the back squat exercise with set 2 in the ST protocol when compared to SW + ST (*p* = 0.037) and FR + ST (*p* = 0.012) as well as in set 3 when compared to FR + ST (*p* < 0.001). The bench press 45° exercise presented higher maximum repetitions in the ST protocol with set 2 when compared to FR + ST (*p* = 0.012) and with set 3 in the FR + ST (*p* < 0.001) and SW + ST (*p* = 0.024) protocols. The front squat exercise presented greater maximum repetitions with ST with set 2 when compared to FR + ST (*p* = 0.030) and AE + ST (*p* = 0.045) and with set 3 in the FR + ST (*p* < 0.001), AE + ST (*p* = 0.004), SW + ST (*p* = 0.037), and SE + ST (*p* = 0.008) protocols. In addition, a greater number of repetitions was performed in the lat pull-down exercise throughout set 2 in the ST protocol when compared to FR + ST (*p* = 0.006) and AE + ST (*p* = 0.037) as well as in set 3 when compared to FR + ST (*p* < 0.001), SW + ST (*p* = 0.019), and SE + ST (*p* = 0.045). Furthermore, more repetitions were performed in the leg press with set 2 in the ST protocol when compared to FR + ST (*p* = 0.045) and SE + ST (*p* = 0.012) as well as in set 3 when compared to FR + ST (*p* = 0.003), AE + ST (*p* = 0.045), SW + ST (*p* = 0.001), and SE + ST (*p* = 0.005). The shoulder press exercise presented elevated maximum repetitions in the ST protocol with set 2 when compared to the FR + ST (*p* = 0.019) protocol and with set 3 in the FR + ST (*p* = 0.006), SW + ST (*p* = 0.019), and AE + ST (*p* = 0.019) protocols. Finally, a higher number of repetitions was performed in the leg extension throughout set 2 in the ST protocol when compared to FR + ST (*p* = 0.004) and SW + ST (*p* = 0.012) as well as in set 3 when compared to FR + ST (*p* < 0.001), AE + ST (*p* = 0.019), SW + ST (*p* = 0.001), and SE + ST (*p* = 0.019) (all maximum repetitions details presented in Table 3).

### 3.3. Fatigue Index

The bench press presented a significantly higher fatigue index with ST when compared to the FR + ST (21.8%, *p* = 0.010) and SW + ST (14.1%, *p* = 0.016) protocols. Similar responses were observed with the back squat, which showed a significantly higher fatigue index with ST compared to FR + ST (29.2%, *p* = 0.001); AE + ST exceeded FR + ST (25.9%, *p* = 0.006) as well. With bench press 45°, the only significant difference showed a higher fatigue index with ST compared to FR + ST (24.9%, *p* = 0.001). The front squat exercise demonstrated a higher fatigue index with ST versus FR + ST (46.3%, *p* < 0.001), SE + ST (26.7%, *p* = 0.019), and AE + ST (26.7%, *p* = 0.030). With the lat pull-down exercise, ST was only significantly greater than FR + ST protocols (46.3%, *p* = 0.005). With the leg press fatigue index, ST significantly exceeded SW + ST (20.4%, *p* = 0.010) and SE + ST (20.9%, *p* = 0.037). For the shoulder press, ST was only significantly higher than the FR + ST fatigue index (25.0%, *p* = 0.045). Finally, leg extension promoted a significantly higher fatigue index with ST compared to FR + ST (20.7%, *p* = 0.008), SW + ST (25.0%, *p* = 0.012), and AE + ST (25.0%, *p* = 0.037) (all fatigue index results presented in Table 3).

### 3.4. Blood Pressure

No significant reduction was observed in systolic and diastolic BP (Table 4) for any protocol or exercise, although the effect size magnitudes ranged from trivial to large (Table 4).

## 4. Discussion

The purpose of the present study was to examine the acute effects of different pre-ST strategies on total training volume, maximum repetition performance, fatigue index, and BP responses in recreationally strength-trained women. The primary result indicates that higher maximum repetitions, total training volume, and resistance to fatigue were observed when ST was performed without any pre-ST strategies. These findings align with the study conducted by Da Silva Telles et al. [3], which also reported that isolated ST with ischemic preconditioning led to a higher number of repetitions compared to other pre-ST strategies. While Da Silva Telles et al. [3] is the only study with a similar experimental design, the present study is the first to compare various pre-ST strategies on muscular (total training volume and resistance to fatigue) and BP response.

The muscular results found partial support for the first hypothesis, which indicated that not using pre-ST strategies would allow for higher maximum repetitions and total training volume. Monteiro et al. [24] suggested that fewer repetitions would result due to the fatigue associated with reduced rest interval or when combining other exercises. Typically, the second exercise in an ST sequence would be expected to exhibit a greater degree of fatigue, as non-local muscle fatigue can adversely affect the performance of previously non-exercised muscles [25,26,27].

Additionally, the total training volume was reduced under all experimental protocols when compared to ST performed alone (Table 3). The resistance to fatigue reduction was expected, as hypothesized in this study, due to residual fatigue caused by the inclusion of another activity as a pre-strength training strategy. The finding of a lower fatigue index was related to the lower maximum repetition performance and total training volume. The reduction in the total training volume may adversely affect the hypertrophic training response given that hypertrophy appears to be volume dependent. These results argue against the inclusion of other pre-ST strategies.

While non-local muscle fatigue has been attributed to physiological mechanisms, such as central nervous system inhibition, and psychological factors like the strength energy model (where prior fatiguing exercise affects subsequent motivation, focus, and attention), there is also evidence of central nervous system potentiation or excitation. For instance, studies by Halperin et al. [26], Aboodarda et al. [28], and Šambaher et al. [29] have demonstrated increased spinal and cortical excitability in contralateral muscles following unilateral fatiguing ST exercises. Aboodarda et al. [28] found enhanced spinal excitability in contralateral knee extensors after fatiguing unilateral elbow flexors, and Šambaher et al. [29] reported similar results with cervico-medullary evoked potentials. Furthermore, Aboodarda et al. [28,30] showed increased cortical excitability in contralateral elbow flexors after fatiguing unilateral elbow flexors and knee extensors, respectively. Overall, the impact of an exercise on subsequent performance is influenced by a delicate balance between fatiguing and potentiating mechanisms [31].

Similarly, active pre-ST protocols tend to generate higher fatigue, as reported by Monteiro et al. [24], who observed a reduction in resistance to fatigue when subjects performed 60 (FR60) and 120 (FR120) minutes of FR. However, Santana et al. [32] contradicted the previous study by reporting an increase in the total training volume when FR was performed in combination with ST (agonist foam rolling, antagonist foam rolling, and paired (agonist/antagonist) foam rolling) when compared to the traditional ST. This presented scientific literature conflict highlights an important gap in regard to total training volume when strength training was performed in combination with FR.

Several strategies have been identified as intervening in the behavior of force production during different exercises. For example, SE seems to have an impact on changing the musculotendinous unit’s length as well as reducing muscle tone due to lower activation of muscle spindles, inhibiting their motor responses, and even modifying the overlapping arrangement of contractile filaments in a way that impairs their sliding and drag, directly interfering with force generation [33]. In the same way, FR and AE can compromise the performance of strength due to increased fatigue or the use of energy substrates that are intensity dependent, respectively. Furthermore, crosstalk mechanisms between pgc1-α and mTOR pathways also compromise strength training adaptations as a function of aerobic activity [34,35]. On the other hand, SW can be considered a potentiating strategy of strength performance through the post-activation potential involving peripheral mechanisms, such as facilitating myosin phosphorylation, and central mechanisms, such as greater motor neuron excitability, and an increase in the number of recruited motor units [36]. Thus, it is observed that pre-ST strategies can have different impacts on muscular responses; thus, it is fitting to analyze different interventions in this sense to observe the outcome.

The BP results found partial support for the second hypothesis, which indicated that multi-joint with large muscle volume exercises may induce previously clinically significant reductions in systolic BP values. Figueiredo et al. [37] have indicated that ST with a higher total training volume seems to be more efficient in decreasing systolic BP in young adults. The neural reflex via the activation of chemical and mechanical receptors activates the afferent pathway to the nucleus of the solitary tract, which stimulates the sympathetic system while inhibiting the parasympathetic system [12]. Thus, large muscle groups can increase the absolute BP values [38], a phenomenon not observed in the present study. For example, Halliwill [14] hypothesizes the local metabolic effect as a PEH mechanism, which has a vasodilator and alpha-adrenergic receptor inhibitory action. These effects become more important than simple reductions in cardiac output given the fact that reductions in cardiac output can be offset by a post-exercise reduction, exercise load, and increased myocardial contractility. It is, therefore, pertinent to hypothesize a possible effect of muscle mass size on the local metabolite production, which supports the results of the present study that uses principally multi-joint exercises and indicates a large effect size post-exercise magnitude (FR + ST, SW + ST, SE + ST, and SE + ST).

Effect sizes indicated a clinical reduction with a large magnitude in systolic BP for the FR + ST (Post-50: ∆ = −5.00 mmHg) protocol when compared to the baseline (Table 4). The findings observed in the FR + ST protocol is consistent with those presented by Monteiro et al. [39]. In their study, the authors also reported PEH in systolic BP for FR at Post-60 (*p* = 0.020; effect size = −2.14) and in ST + FR at Post-50 (*p* = 0.001; effect size = −2.03) and Post-60 (*p* < 0.001; effect size = −2.38) time points. Similar results were described by Monteiro et al. [38] when observed PEH in systolic BP after a massage alone (Post-50: *p* = 0.011; effect size = −2.61; ∆ = −4.0 mmHg; Post-60: *p* = 0.011; ES = −2.74; ∆ = −4.0 mmHg) but not performed after ST. Although using different techniques, the literature consistently demonstrates similar effects between FR and massage techniques. In a systematic review with a meta-analysis, Liao et al. [40] found that both FR and massage techniques significantly contributed to reductions in systolic (−7.39 mmHg; effect size = −0.728) and diastolic (−5.04 mmHg; effect size = −0.334) BP. Therefore, incorporating FR before and after ST appears to be a valuable clinical approach for PEH.

Different physiological mechanisms seem to justify the results found both in the present study and in the previous literature. First, Okamoto et al. [8] reported higher levels of nitric oxide following FR, suggesting a greater vasodilatory effect that may contribute to the reduction in BP. Similarly, Hotfiel et al. [9] observed increased local arterial perfusion in the lateral thigh region after FR, which they associated with vasodilation resulting from the elevation of nitric oxide levels following FR. Second, White and Raven [41] indicated that during exercise, there is a reversal in the action of the autonomic nervous system in the control of cardiac activity in an attempt to maintain homeostasis, thus reducing vagal control. However, Farinatti et al. [41]’s systematic review indicates that relatively prolonged changes (≥30 min) in BP post-ST were shown to be inversely related to changes in sympathetic and directly related to changes in parasympathetic outflow; that is, PEH was systematically concomitant to increased sympathetic and decreased parasympathetic modulation, irrespective of the BP status of samples or study design.

Forjaz et al. [42], in a classic study, demonstrated that continuous low-intensity AE was efficient in providing PEH, which would be related to reduced sympathetic activity as well as the vasodilator effect of local metabolites. Inami et al. [43] observed that systolic BP was transiently higher during SE and returned to baseline immediately post-intervention. The present results followed those found by Inami et al. [43] when observing that only the SW + ST (effects size = 0.94) and AE + ST (effect size = 1.96) protocols had a large magnitude in Post-10. A potential mechanism to explain the PEH after SE was reported by Kruse and Scheuermann [44], who indicate a reduction in blood flow by a reduction in the diameter of blood vessels through a mechanical obstruction generated by muscle contractions and also by the nutrient supply. Kruse and Scheuermann [45] observed mechanical vascular deformation at the beginning of stretching combined with an increase in the stimulation of group III afferent fibers, which initiates a cascade of events, resulting in peripheral vasodilation and an increase in heart rate, cardiac output, BP, and blood flow.

Finally, the results of this study offer interesting practical applications. While all pre-ST interventions indicated a clinically significant reduction in BP values (large effect size), the FR + ST and SW + ST conditions showed significant decreases in fatigue index, indicating a decline in muscle performance. Therefore, the results suggest that the AE + ST and SE + ST conditions may be more efficient as pre-ST strategies.

A point to consider when interpreting the findings in this study is the self-paced FR and ST either within or between individuals. This can be considered as both a limitation and a strength of this design. Specifically, the lack of control reduces the internal validity of the results, as the number/duration of each roll could influence the outcome. The freedom to choose the pace and duration of each repetition enhances the ecological validity and generalizability of the findings, as it better represents real-life training scenarios and suggests a more robust influence of strength training on muscular performance and blood pressure responses rather than having to meet tightly controlled parameters to elicit a response. The participants in the current study were all women. It has been well documented that women generally exhibit lower relative muscle strength compared to men during dynamic contractions. The behavior of cardiovascular parameters at rest does not appear to differ across the various phases of the menstrual cycle. Despite PEH being a resting phenomenon—occurring during recovery from exertion—it is worth considering that hemodynamic parameters could exhibit distinct responses in this context [45]. However, specifically regarding PEH, even with a potential difference in magnitude, the phases of the menstrual cycle do not act as a binary predictor of this variable. In other words, none of the phases seem to prevent PEH from occurring if the protocol has the potential to induce this phenomenon. The menstrual cycle’s different phases can indeed influence responses, as reported by Queiroz et al. [46]. Their study indicated that the occurrence of menstrual bleeding may affect the magnitude of BP decrease but not the overall occurrence of this phenomenon [46]. While it is essential to exercise caution when generalizing these findings, it is important to note that the primary focus of the present study was not to analyze mechanisms but rather to examine the BP response. However, we intentionally chose not to control for this factor because we believe it better reflects real-world scenarios and enhances the ecological validity of our results.

Consequently, the fact that the participants were women is not likely to pose significant issues when extrapolating the results to men, as the key difference between sexes lies in the underlying mechanisms rather than the blood pressure responses [46]. The results presented were not sensitive enough to demonstrate significant differences in the variable blood pressure; however, they do show medium effect size values. The observation of a medium effect size in the control condition, although initially unexpected, can be reasonably explained by well-established physiological factors. Even without active intervention, factors such as the placebo effect and natural adaptive physiological processes can lead to improvements or noticeable changes in participants’ conditions [47,48,49].

## 5. Conclusions

We found decreases in maximum repetitions, resistance to fatigue, and total training volume when warm-up strategies were performed before ST. Thus, the pre-ST strategies of FR, SW, AE, and SE are not performed if the main objective of the ST protocol is improvements in muscular performance (for example, the maintenance of the number of repetitions). However, these strategies indicated a clinical reduction in BP with a large and meaningful magnitude (effect size) in recreationally strength-trained women with normal to elevated BP. The results of this investigation may help to influence decision-making by practitioners who desire to elicit a PEH response in both subjects with normal BP and hypertension. In addition, while investigating chronic changes in PEH as a response to these interventions was beyond the scope of this current investigation, there is ample data to suggest that strength training can be a powerful tool to improve blood pressure. Practitioners who are looking to influence blood pressure parameters in their clients may wish to explore incorporating strength training in their plan of care.

## Figures and Tables

**Table 1:** Subject’s load characteristics.

Variable	Mean ± Standard Deviation
Bench Press 10 RM Test (kg)	36.33 ± 3.98
Bench Press 10 RM Retest (kg)	36.17 ± 3.46
Bench Press 10 RM ICC	0.824 (CV% = 12.23)
Back Squat 10 RM Test (kg)	59.33 ± 10.49
Back Squat 10 RM Retest (kg)	60.50 ± 13.35
Back Squat 10 RM ICC	0.946 (CV% = 21.01)
Bench Press 45° 10 RM Test (kg)	34.00 ± 4.18
Bench Press 45° 10 RM Retest (kg)	33.83 ± 3.01
Bench Press 45° 10 RM ICC	0.555 (CV% = 13.68)
Front Squat 10 RM Test (kg)	44.83 ± 7.79
Front Squat 10 RM Retest (kg)	43.33 ± 7.69
Front Squat 10 RM ICC	0.861 (CV% = 19.98)
Lat Pull-Down 10 RM Test (kg)	42.92 ± 8.38
Lat Pull-Down 10 RM Retest (kg)	41.6 ± 6.85
Lat Pull-Down 10 RM ICC	0.908 (CV% = 8.37)
Leg Press 10 RM Test (kg)	233.33 ± 47.67
Leg Press 10 RM Retest (kg)	231.67 ± 36.64
Leg Press 10 RM ICC	0.9230 (CV% = 13.11)
Shoulder Press 10 RM Test (kg)	35.42 ± 4.75
Shoulder Press 10 RM Retest (kg)	35.83 ± 5.47
Shoulder Press 10 RM ICC	0.920 (CV% = 13.11)
Leg Extension 10 RM Test (kg)	80.83 ± 11.65
Leg Extension 10 RM Retest (kg)	81.25 ± 10.69
Leg Extension 10 RM ICC	0.972 (CV% = 13.17)

Legend: 10 RM = ten repetition maximum; ICC = Intraclass Coefficient for 10 RM test and Retest; CV = Coefficient of Variation.

**Table 2:** Total training volume throughout each exercise and conditions.

	Protocols				
Exercises	ST	FR + ST	SW + ST	AE + ST	SE + ST
Bench Press ^+^	2550 (2360–2895)	2172 (2017–2403) *	2172 (2080–2397.5) *	2217 (2097.5–2719.5)	2311 (2166–2420.5) *
Back Squat ^+^	3080 (2657.5–3227.5)	2465 (2262.5–2860) *	2530 (2257.5–3117.5) *	2860 (2447.5–3090)	2725 (2447.5–3142.5)
Bench Press 45° ^++^	2377.5 ± 374.97	1955 ± 258.77 *	2056.5 ± 354.9 *	2109.5 ± 2776 *	2165.33 ± 311.47 ^#^
Front Squat ^+^	2094 (1981–2447)	1812 (1528.5–1963.5) *	1902 (1685.5–2189.5) *	1818 (1632–2136) *	1877 (1692–2315.5) *
Lat Pull-Down ^++^	2347.5 (2191.25–2683.75)	2017.5 (1780–2197.5) *	2062.5 (1927.5–2307.5) *	2210 (1940–2250) *	2185 (1807.5–2362.5) *
Leg Press ^++^	6810 (5600–7995)	5740 (4800–6945) *	5720 (5010–6500) *	5880 (5220–7210) *	5740 (4800–6665) *
Shoulder Press ^++^	997.5 (976.25–1156.87)	842.5 (743.75–1023.75) *	880 (770–1035) *	918.75 (813.75–1059.37) *	915 (817.5–1050)
Leg Extension ^++^	2280 (2115–2485)	1865 (1697.5–2160) *	2007.5 (1760–2197.5) *	2058.75 (1852.5–2295)	1988.75 (1825–2360)

Legend: ST = strength training isolated; FR + ST = foam rolling warm-up followed by strength training; SW + ST = specific warm-up followed by strength training; AE + ST = aerobic exercise followed by strength training; SE + ST = stretching exercise followed by strength training. ^+^ Values represented by median and interquartile range. ^++^ Values represented by mean and standard deviation. * Significant difference (*p* < 0.05) in relation to the ST condition; ^#^ significant difference (*p* < 0.05) in relation to the FR + ST protocol.

**Table 3:** Maximum repetition performance and fatigue index values for each exercise and conditions (median (interquartile range)).

	Set 1	Set 2	Set 3	FI (%)
BENCH PRESS				
ST	10 (9.25–10.75)	10 (9–10.75)	9 (9–10)	90 (84.99–100)
FR + ST	9 (8.25–10)	8 (8–9) ^@^	7 (6–8) *^@^	73.86 (63.54–86.66) ^@^
SW + ST	9.5 (9–10)	8 (7.25–8.5) ^#@^	7.5 (7–8) *^@^	78.89 (70–86.66) ^@^
AE + ST	9.5 (9–10)	9 (8–9.75)	8 (7–9) *	85.35 (71.94–100)
SE + ST	9 (9–10)	8 (7.25–9) ^@^	8 (7–9) *	80 (77.78–100)
**BACK SQUAT**				
ST	10 (9.25–10.75)	9.5 (9–10)	9 (9–10)	90.45 (90–100)
FR + ST	10 (9–10)	8 (7–8.75) ^@^	7 (5.25–7) *^@^	70 (60–77.78) ^@&^
SW + ST	9 (8–9.75)	8 (7–9) ^@^	7.5 (7–9) *	87.5 (77.78–97.5)
AE + ST	9 (9–10)	9 (8–9)	8 (7–9.75)	88.19 (80–100)
SE + ST	9.5 (9–10)	9 (8.25–10)	8 (7–8.75)	80 (77.78–97.22)
**BENCH PRESS 45°**				
ST	10 (9–10)	10 (9–10)	9.5 (9–10)	95.45 (90–108.33)
FR + ST	9 (8–10)	8 (7.25–8.75) ^@^	7 (6–7.75) *^@^	76.39 (71.25–80) ^@^
SW + ST	9 (8–9.75)	8 (8–9)	7.5 (7–9) *^@^	88.19 (71.25–100)
AE + ST	9 (9–10)	8 (8–9)	8 (7.25–9) *	88.89 (80–100)
SE + ST	9.5 (9–10)	9 (8–10)	8 (7–8.75)	80 (77.78–97.22)
**FRONT SQUAT**				
ST	10 (9.25–10.75)	10 (9.25–10.75)	9.5 (9–10)	100 (83.86–107.5)
FR + ST	9 (8.25–10)	8 (7–9) ^@^	7 (6–7) *^@^	72,5 (67.5–79.44) ^@^
SW + ST	9 (9–10)	8.5 (8–9)	8 (7–8.75) *^@^	83.75 (77.78–90)
AE + ST	9.5 (8.25–10)	8 (7.25–9) ^@^	7 (7–8) *^@^	78.89 (70–87.5) ^@^
SE + ST	10 (9–10)	95 (8–10.75)	7.5 (7–8.75) *^$@^	78.89 (70–95) ^@^
**LAT PULL-DOWN**				
ST	10 (9–10.75)	10 (9–10)	9 (9–10)	100 (83.86–100)
FR + ST	9.5 (9–10)	8 (7.25–9) ^@^	6.5 (6–7.75) *^@^	68.33 (56.67–79.44) ^@^
SW + ST	9.5 (9–10)	8.5 (8–9)	7 (7–8) *^@^	78.89 (70–88.89)
AE + ST	9.5 (9–10)	8 (7.25–9) ^@^	8 (7.25–9)	88.19 (80–100)
SE + ST	10 (9–10)	8 (8–10)	8 (7–8) *^@^	80 (77.78–95)
**LEG PRESS**				
ST	10 (9–10)	9.5 (9–10)	9 (9–10)	95 (90–111.11)
FR + ST	9 (8.25–10)	8 (8–8.75) ^@^	7 (6.25–8) *^@^	77.78 (67.5–100)
SW + ST	9 (9–10)	8 (7.25–9)	7 (7.25–8) *^@^	78.89 (71.25–85.62) ^@^
AE + ST	9.5 (9–10)	8 (7.25–9)	8 (7–8) *^@^	80 (77.78–100)
SE + ST	9 (9–10)	8 (7–8.75) ^@^	7.5 (7–8) *^@^	83.75 (71.94–88.89) ^@^
**SHOULDER PRESS**				
ST	10 (9–10)	9 (9–10)	9 (9–10)	100 (90–100)
FR + ST	9 (8.25–10)	8 (7–8) ^@^	7.5 (6–8) *^@^	80 (66.67–88.54) ^@^
SW + ST	9 (9–10)	8 (7–8.75) ^#^	7 (7–8) *^@^	78.89 (70–89.72)
AE + ST	9.5 (9–10)	8 (7.25–9)	7 (7–8.75) *^@^	77.78 (70–100)
SE + ST	9.5 (9–10)	9 (7.25–10)	7.5 (7–9.5) *	78.89 (75.69–100)
**LEG EXTENSION**				
ST	10 (9–10)	10 (9–10)	9.5 (9–10)	100 (92.5–100)
FR + ST	9 (8–10)	8 (7–8) ^@^	7 (6–8) *^@^	82.85 (70–97.22) ^@^
SW + ST	9 (8.25–10)	8 (7–9) ^@^	7 (7–8) *^@^	80 (70–88.54) ^@^
AE + ST	9 (9–10)	8.5 (7.25–9)	8 (7–8) *^@^	80 (77.78–97.22)
SE + ST	9.5 (9–10)	8 (7.25–10)	8 (7–8) *^@^	80 (75.69–88.89) ^@^

Legend: ST = strength training isolated; FR + ST = foam rolling warm-up followed by strength training; SW + ST = specific warm-up followed by strength training; AE + ST = aerobic exercise followed by strength training; SE + ST = stretching exercise followed by strength training. FI = fatigue index. ^#^ significant difference between set 1 and set 2 (*p* < 0.05). * significant difference between set 1 and set 3 (*p* < 0.05). ^$^ significant difference between set 2 and set 3 (*p* < 0.05). ^@^ significant difference for ST (*p* < 0.05). ^&^ significant difference for AE + ST (*p* < 0.05).

**Table 4:** Blood pressure (means, standard deviation, and effects sizes) throughout all exercises and conditions.

Protocol	Baseline	Post-10 min	Post-20 min	Post-30 min	Post-40 min	Post-50 min	Post-60 min
		Systolic Blood Pressure					
CC	118.33 ± 4.89	114.83 ± 4.22	115.67 ± 4.58	115.50 ± 4.98	115.33 ± 5.42	115.33 ± 5.61	115.83 ± 5.49
		−0.76 (Medium)	−0.56 (Medium)	−0.57 (Medium)	−0.58 (Medium)	−0.57 (Medium)	−0.48 (Small)
ST	121.83 ± 3.95	124.67 ± 3.85	122.33 ± 3.89	121.83 ± 3.95	119.67 ± 3.50	119.17 ± 4.04	118.50 ± 4.76
		0.72 (Medium)	0.12 (No Effect)	0.00 (No Effect)	−0.57 (Medium)	−0.66(Medium)	−0.76 (Medium)
FR + ST	116.17 ± 6.52	117.67 ± 6.43	115.67 ± 5.38	113.33 ± 6.51	11,167 ± 5.65	111.17 ± 5.87	111.67 ± 5.96
		0.23 (Small)	−0.08 (No Effect)	−0.43 (Small)	−0.73 (Medium)	−0.80 (Large)	−0.72 (Medium)
SW + ST	121.33 ± 4.46	125.83 ± 5.08	123.50 ± 4.36	120.83 ± 4.47	118.33 ± 4.16	117.83 ± 4.22	117.17 ± 5.22
		0.94 (Large)	0.49 (Small)	−0.11 (No Effect)	−0.69 (Medium)	−0.80 (Large)	−0.85 (Large)
AE + ST	122.83 ± 2.62	127.83 ± 2.48	123.83 ± 3.35	121.67 ± 2.23	120.50 ± 2.11	119.33 ± 2.74	118.83 ± 3.01
		1.96 (Large)	0.33 (Small)	−0.47 (Small)	−0.98 (Large)	−1.30 (Large)	−1.42 (Large)
SE + ST	116.83 ± 5.08	115.83 ± 6.24	113.50 ± 4.60	112.00 ± 4.67	111.83 ± 4.86	111.67 ± 4.42	112.00 ± 3.72
		−0.17 (No Effect)	−0.68 (Medium)	−0.99 (Large)	−1.00 (Large)	−1.08 (Large)	−1.09 (Large)
		**Diastolic Blood Pressure**					
CC	78.50 ± 4.10	78.83 ± 3.66	79.00 ± 3.77	78.83 ± 3.86	78.83 ± 3.76	78.50 ± 3.73	78.83 ± 3.86
		0.08 (No Effect)	0.12 (No Effect)	0.08 (No Effect)	0.08 (No Effect)	0.00 (No Effect)	0.08 (No Effect)
ST	80.00 ± 2.70	80.50 ± 2.28	80.33 ± 2.39	80.17 ± 2.62	80.00± 2.56	80.50 ± 2.58	80.50 ± 2.71
		0.20 (Small)	0.12 (No Effect)	0.06 (No Effect)	0.00 (No Effect)	0.18 (No Effect)	0.18 (No Effect)
FR + ST	77.67 ± 5.45	78.17 ± 5.69	77.17 ± 5.42	77.33 ± 4.77	77.83 ± 5.42	77.83 ± 5.49	78.00 ± 5.72
		0.08 (No Effect)	−0.09 (No Effect)	−0.06 (No Effect)	0.02 (No Effect)	0.02 (No Effect)	0.05 (No Effect)
SW + ST	80.33 ± 2.81	80.17 ± 2.33	80.17 ± 2.48	80.50 ± 2.71	80.33 ± 2.39	80.17 ± 2.33	80.17 ± 2.76
		−0.06 (No Effect)	−0.06 (No Effect)	0.06 (No Effect)	0.00 (No Effect)	−0.06 (No Effect)	−0.05 (No Effect)
AE + ST	78.50 ± 4.10	78.50 ± 4.10	78.50 ± 4.10	78.67 ± 3.75	78.17 ± 4.04	78.50 ± 4.01	78.67 ± 3.75
		0.00 (No Effect)	0.00 (No Effect)	0.04 (No Effect)	−0.08 (No Effect)	0.00 (No Effect)	0.04 (No Effect)
SE + ST	78.50 ± 4.10	78.17 ± 4.30	78.33 ± 3.98	78.83 ± 4.04	78.50 ± 4.52	78.50 ± 4.68	78.67 ± 4.38
		−0.07 (No Effect)	−0.04 (No Effect)	0.08 (No Effect)	0.00 (No Effect)	0.00 (No Effect)	0.04 (No Effect)

Legend: CC = control condition; ST = strength training isolated; FR + ST = foam rolling warm-up followed by strength training; SW + ST = specific warm-up followed by strength training; AE + ST = aerobic exercise warm-up followed by strength training; SE + ST = stretching exercise warm-up followed by strength training.

## Data Availability

Data are contained within the article.
